# Expression of ERBB3 binding protein 1 (EBP1) in salivary adenoid cystic carcinoma and its clinicopathological relevance

**DOI:** 10.1186/1471-2407-12-499

**Published:** 2012-10-30

**Authors:** Jian Sun, Yixi Luo, Zhen Tian, Liang Gu, Shu Chi Xia, Youcheng Yu

**Affiliations:** 1 Department of Stomatology, Zhongshan Hospital, Fudan University, Shanghai, 200032, China; 2 Department of Pathology, the Ninth People’s Hospital, School of Medicine, Shanghai Jioa Tong University, Shanghai, 200011, China

## Abstract

**Background:**

ERBB3 binding protein 1 (***EBP1***) gene transfer into human salivary adenoid cystic carcinoma cells has been shown to significantly inhibit cell proliferation and reduce tumor metastasis in mouse models. In the current study, to evaluate if EBP1 is a novel biomarker capable of identifying patients at higher risk of disease progression and recurrence, we examined the EBP1 expression profile in adenoid cystic carcinoma (ACC) patients and analyzed its clinicopathological relevance. To understand the underlying anti-metastatic mechanism, we investigated if EBP1 regulates invasion-related molecules.

**Methods:**

We performed immunohistochemical analysis on 132 primary adenoid cystic carcinoma and adjacent non-cancerous tissues using commercial EBP1, MMP9, E-cadherin and ICAM-1 antibodies. Results were correlated to clinicopathological parameters, long-term survival and invasion-related molecules by statistical analysis. Cell motility and invasiveness of vector or wild-type *EBP1*-transfected ACC-M cell lines were evaluated using wound healing and Boyden chamber assays. MMP9, E-cadherin and ICAM-1 proteins in these cell lines were detected using western blot assay.

**Results:**

The expression of EBP1 was significantly higher in non-cancerous adjacent tissues compared with corresponding cancer tissues. The intensity and percentage of cells that reacted with EBP1 antibodies were significantly higher in cases with tubular pattern than those with solid pattern (*P*<0.0001). We also found adenoid cystic carcinoma with local lymphatic metastasis had significantly lower EBP1 expression than ACC with no local lymphatic node metastasis (*P*<0.0001). Similar findings were observed in ACC with lung metastasis compared with cases with no lung metastasis (*P*<0.0001), in particular, in cases with perineural invasion compared with cases with no perineural invasion (*P*<0.0001). Furthermore, a decrease in EBP1 expression was positively associated with a reduction in overall survival of ACC patients. Of note, EBP1 inhibits migration and invasiveness of ACC cells by upregulating E-cadherin but downregulating MMP9. In clinical adenoid cystic carcinoma patients, higher EBP1 expression was positively correlated with E-cadherin levels (*P*<0.001) but negatively correlated with MMP9 expression (*P*=0.0002).

**Conclusions:**

EBP1 expression is reduced in adenoid cystic carcinoma, indicating unfavorable prognosis of ACC patients. Its regulation of MMP9 and E-cadherin protein levels suggests a critical therapeutic potential.

## Background

Adenoid cystic carcinoma (ACC) of the major and minor salivary glands is a relatively rare epithelial tumor [1-3]. However, ACCs are highly aggressive neoplasms with a predilection for perineural infiltration, which partially explains the tendency for local recurrence [1,2]. Unlike squamous cell carcinoma in the head and neck, ACC often spreads systemically to lungs and bone, leading to a 20-year survival rate of only about 11% [1-3]. Therefore, it will be of great clinical value to identify the molecular events associated with the development and progression of ACC for early detection and prognosis, in particular the targets for therapeutic treatment [4].

ERBB3 binding protein 1 (EBP1) is the human homologue of a previously identified cell cycle-regulated mouse protein p38-2G4 [5]. EBP1 is a conserved molecule across species with multiple roles in cell proliferation and differentiation [6-10]. In our previous study, we demonstrated that wild-type *EBP1* gene transfer into human salivary adenoid cystic carcinoma cells significantly inhibits cell proliferation in *in vitro* assays, and most importantly, reduces tumor metastatic potential in an experimental metastatic mouse model [11], consistent with its inhibitory property identified in cancers of glandular epithelial origin such as prostate [12,13] and breast [14]. In this study, we investigated the EBP1 expression profile in adenoid cystic carcinoma patients to evaluate if EBP1 is a novel biomarker capable of identifying patients at higher risk of disease progression and recurrence. Our results suggest that EBP1 immunoreactivity inversely correlates with local invasion and distant spread of adenoid cystic carcinomas. Patients with lower EBP1 levels had poorer long-term survival than those with higher EBP1 expression.

## Methods

### Patients and specimens

Formalin-fixed, paraffin-embedded specimens were randomly selected from the primary tumor and adjacent non-cancerous tissues of 66 patients suffering from operable ACCs and thus undergoing curative surgery at the Ninth People’s Hospital, JiaoTong University from 2004 to 2007. All archival blocks were stored at room temperature in a modern centrally air-conditioned histology laboratory. None of the cases had received pre-operative chemotherapy or radiotherapy. Histopathological grading of ACCs was performed according to WHO classification [15], and tumor staging was based on the tumor-node-metastasis (TNM) system [16]. Medical records and prognostic follow-up data were obtained from the patient database completed by physicians and data managers after each patient visit. Independent Ethics Committee of Shanghai Ninth People’s Hospital affiliated to Shanghai JiaoTong University, School of Medicine approved the study protocol (Number 201287).

### Immunohistochemistry (IHC)

Hematoxylin and eosin (H&E) sections were analyzed for the presence of tumors. Sections containing the highest number of tumors were selected for each patient. The corresponding tissue blocks were then recut into 5-μm thick sections and mounted on charged slides. The sections were deparaffinized in toluene and rehydrated in a gradient series of ethanol. Endogenous peroxides were quenched by treatment with 0.3% H_2_O_2_/methanol for 30 min at room temperature. Antigen retrieval was accomplished by microwave heating at 90°C for 15 min in citrate buffer (10 mM, pH 6.0). Slides were incubated with rabbit polyclonal anti-EBP1 antibody (EMD Millipore Corporation, Billerica, MA, USA), MMP9 rabbit polyclonal antibody, E-cadherin rabbit mAb, ICAM-1 rabbit polyclonal antibody (Cell Signaling Technology, Inc., Danvers, MA, USA) following immunohistochemistry protocols provided by the manufacturers. Specific staining was detected by applying the Vectastain Elite ABC Kit (Vector Laboratories). For negative control, tissues were incubated with non-immunized purified rabbit IgG (EMD Millipore Corporation, Billerica, MA, USA).

### Evaluation of immunostaining

Two independent pathologists examined randomly selected 10 fields per stained section, and two sections for each specimen using light microscope at 40× magnification. The percentage of cells with positive EBP1staining was semi-quantitatively assessed using a four-tiered scoring system: negative (−), <5% positive cells; intermediate (+), 5–25%; moderate (++), 25–50% and strong (+++), 50–100% of cells stained.

### Cell culture

The generation of ACC-M cell lines stably transfected with EBP1 cDNA or a vector control was previously described [11]. We used ACC-M, ACC-M0 (ACC-M-pcDNA3.1) and ACC-M1 (ACC-M-*EBP1*-1μg) as Control, Vector and ebp1.

### Western blotting

Western blot analysis was performed as previously described [11]. Antibody against GADPH was purchased from Sigma. Specific antibodies for EBP1, MMP9, ICAM-1 and E-cadherin were the same as those used for immunohistochemistry analysis. We followed the protocols provided by the manufacturers for the concentration of these antibodies in Western blot analysis.

### Boyden chamber assay

A modified Boyden chamber assay was used to determine cell invasion, as described previously [13]. In brief, culture plate inserts (8-um pore size and 12-mm diameter, Millicell-PCF) were coated with 150 μl PBS containing 10 μg collagen and 1μg fibronectin (BD Bioscience) for 1 h at room temperature before adding cells suspended in 450 μl RPMI 1640 medium with 5% charcoal-striped serum. The bottom wells of the system were filled with 600 μl complete medium. After 24 h in a humidified atmosphere of 5% CO_2_ in air, the inserts were fixed in 10% formalin for 30 min and after washing with PBS, stained with 0.5% crystal violet in 25% methanol for 45 min. Non-migrating cells on the top of the filters were removed with a cotton swab. Cells that had migrated to undersurface of the filter were examined at 20× magnification. The number of cells in three representative areas was counted. Each experiment was performed in triplicate.

### Wound healing assay

The assay was performed as described previously [17]. Briefly, cells were seeded in six-well plates at a density of 5×10^6^ cells/well and grown to confluence. The monolayer culture was then artificially scrape-wounded with a sterile micropipette tip to create a denuded zone (gap) of constant width. After the detached cells were removed with serum-free RPMI 1640, cells that had migrated to the wounded region were observed by Olympus CK-2 inverted microscope and photographed (100× magnification). The resulting images were compiled in Adobe Photoshop. The wound areas were measured by the program Image J ( http://rsb.info.nih.gov.proxy-hs.researchport.umd.edu/ij/). Average migration speed was calculated from the average distance traveled and the time elapsed between images.

### Statistical analysis

Associations were evaluated with Fisher’s exact test, Wilcoxon test, Kruskal Wallis test and Spearman rank correlation test. Survival analysis was carried out using Kaplan–Meier estimates, log rank tests and Cox’s proportional hazards regression analysis. All tests were two-sided, and the significance level was set at 5%. All of the statistical analysis was performed using SPSS 13.0 software (SPSS Inc, Chicago, IL, USA).

## Results

### Immunohistochemical staining of EBP1 expression in ACCs

**Figure 1:**
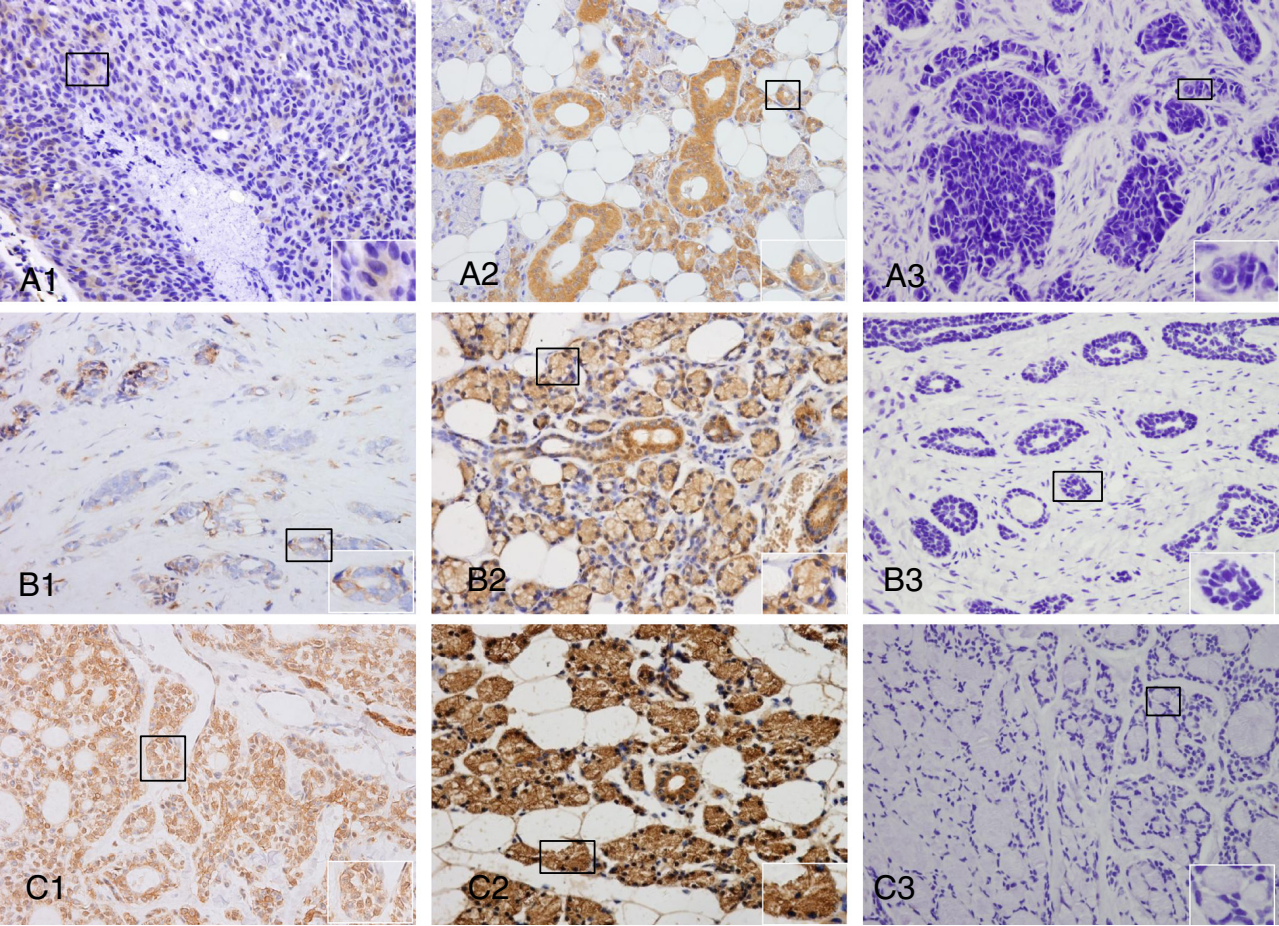
**Detection of EBP1 in ACC and adjacent non-cancerous tissues by immunohistochemical staining.** Representative sections of solid (A1), tubular (B1) and cribriform (C1) patterns of ACCs showing intermediate (+) EBP1 staining (×40). Strongly positive staining (+++) was observed in adjacent non-cancerous tissues (A2, B2 and C2, 40). The same adjacent non-cancerous tissues (A3, B3 and C3) incubated with concentration-matched non-immune rabbit IgG showed no staining. Magnification of relevant parts of the images are included as insets.

To evaluate if EBP1 status is linked to the clinical progression of ACC, immunohistochemistry analysis was performed to examine EBP1 protein expression in 132 paraffin-embedded normal adjacent and carcinoma tissues. Examples of immunohistochemical stains in different types of ACC and adjacent non-cancerous tissues with rabbit polyclonal anti-human EBP1 antibody are shown in Figure 1. In ACC, EBP1 staining was localized predominantly to the cytoplasm of epithelial cells of glands, whereas in the adjacent non-cancerous tissues, abundant EBP1 immunoreactivity was observed in both the cytoplasm and nuclei. Incubation with purified normal rabbit polyclonal IgG did not result in any staining in adjacent non-cancerous tissues, indicating the specificity for EBP1. Among 66 ACC tissues (Table 1), 56 showed positive EBP1 staining (84.9%) with variable staining intensity. Scores were “-” in 10 cases, “+” in 9 cases (13.6%), “++” in 24 cases (36.4%) and “+++” in 23 cases (34.9%). In contrast, the proportion of positive EBP1 staining (98.49%) was significantly higher in non-cancerous adjacent tissues compared with corresponding cancer tissues (*P*=0.0402), with 17 cases (25.8%) scored as “+”, 3 cases (4.6%) as “++” and 45 cases (68.2%) as “+++”, indicating EBP1 is decreased in the progression of ACC.

**Table 1:** Expression of Ebp1 in ACC and matched para-carcinoma tissues

**Group**	**No. of positive (%)**	**++**	**+++**	**No. of negatives (%)**
	**+**			
ACC	9(13.64%)	24(36.36%)	23(34.85%)	10(15.15%)
Para-Ca	17(25.76%)	3(4.55%)	45(68.18%)	1(1.52%)
P value	0.0402			

### Relationship between EBP1 expression and clinicopathological parameters

The results of the immunohistochemical analysis of EBP1 staining intensity were further statistically analyzed to determine the relationship between EBP1 intensity and clinicopathological variables. As shown in Table 2, there was no significant association of EBP1 staining level with age at diagnosis (*P*=0.1597) and gender (*P*=0.6956). However, EBP1 expression status was significantly correlated with histology subtype (*P*=0.00005). The intensity and percentage of cells that reacted with EBP1 antibodies were significantly higher in cases with tubular pattern than that with solid pattern (*P*<0.0001), although there was no significant correlation of EBP1 immunoreactivity in cases with solid pattern compared with that with cribriform pattern (*P*=0.6393) or similarly, tubular *vs* cribriform type (*P*=0.2075). Interestingly, we found ACC with local lymphatic metastasis had a significantly lower percentage of EBP1 expression than ACC with no local lymphatic node metastasis (42% versus 94%, *P*<0.0001). Similar findings were observed in ACC with distant lung metastasis compared with cases with no lung metastasis (50% versus 94%, *P*<0.0001), particularly in cases with perineural invasion compared with cases with no perineural invasion (60.9% versus 95.4%, *P*<0.0001). In addition, in early clinical stages (Table 2, T1–2), 92.8% samples of human ACC tissues examined were immunohistochemically stained with antibody against EBP1 with advanced disease (Table 2, T3–4), and only 70.8% tissues retained EBP1 staining. The reduced or absent EBP1 was inversely associated with higher clinical stage (*P*=0.0235).

**Table 2:** Relationship between Ebp1 expression and clinicopathological features of ACC patients

	**Variables**	**N (%)**	**-**	**+**	**++**	**+++**	**p-value**	
Age at diagnosis	<=50	27	1(3.70%)	6(22.22%)	8(29.63%)	12(44.44%)	0.1597	
	>50	39	9(23.08%)	3(7.69%)	16(41.03%)	11(28.21%)		
Gender	Male	33	4(12.12%)	5(15.15%)	12(36.36%)	12(36.36%)	0.6956	
	Female	33	6(18.18%)	4(12.12%)	12(36.36%)	11(33.33%)	0.6956	
Histologic subtypes	Tubular	38	3(7.89%)	1(2.63%)	15(39.47%)	19(50.00%)	0.0005	TvsS<.0001
	solid	21	5(23.81%)	6(28.57%)	9(42.86%)	1(4.76%)		TvsC0.2075
	cribriform	7	2(28.57%)	2(28.57%)	0(0.00%)	3(42.86%)		SvsC0.6393
Lung metastasis	+	16	8(50.00%)	5(31.25%)	2(12.50%)	1(6.25%)	<0.0001	
	-	50	3(6.00%)	18(36.00%)	12(24.00%)	17(34.00%)		
Lymphatic metastasis	+	14	8(57.14%)	6(42.86%)	0(0.00%)	0(0.00%)	<0.0001	
	-	52	3(5.77%)	17(32.69%)	14(26.92%)	18(34.62%)		
Perineural invasion	+	23	9(39.13%)	8(34.78%)	4(17.39%)	2(8.70%)	0.002	
	-	43	2(4.65%)	15(34.88%)	10(23.26%)	16(37.21%)		
TNMstaging	T1-2	42	3(7.14%)	6(14.29%)	15(35.71%)	18(42.86%)	0.0235	
	T3-4	24	7(29.17%)	3(12.50%)	9(37.50%)	5(20.83%)		

### EBP1 inhibits migration of ACC cells *in vitro*

**Figure 2:**
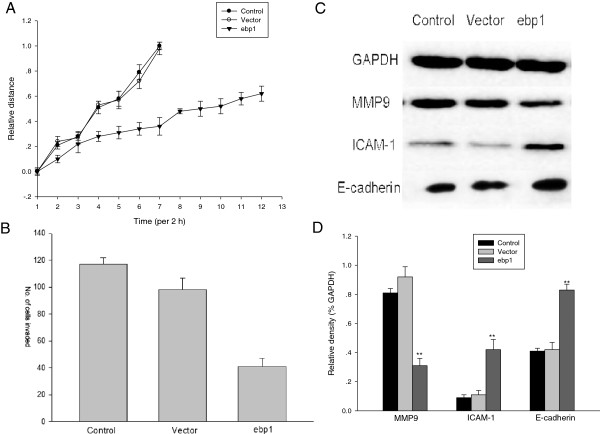
**EBP1 suppresses the motility and invasiveness of ACC-M cells by modulating the expression of invasion-related molecules.A**. Effect of EBP1 on cell migration was investigated using a wound-healing assay, as described in the Materials and Methods. **B**. EBP1 inhibits the invasion of ACC-M cells *in vitro*. Cells that penetrated through the Matrigel to the lower surface of the filter were stained with crystal violet. Quantification of cells in the lower chamber was performed by counting; the mean number of cells that invaded in three representative fields per well is expressed; bars, SD. *P<0.05. Data is representative of three independent experiments. **C**. Protein levels of MMP9, ICAM-1 and E-cadherin in ACC-M cell line stably transfected with pcDNA 3.1 or pcDNA-*EBP1* plasmids were analyzed by western blotting assay, and GAPDH was used as a loading control. **D**. Columns represent relative band densities normalized to GAPDH as imaged in A. **P<0.05.

Cellular migration is one of fundamental features of cancer metastasis. To confirm the anti-metastatic potential of EBP1 based on the relationship between EBP1 expression and clinicopathological parameters, wound-healing assays were first performed to examine the effect of EBP1 on the migratory features of ACC cells. Control cells migrated toward the scratched region, resulting in ‘wound healing’ with a narrow margin. On the other hand, cells transfected with EBP1 cDNA inhibited the migration of cells (Figure 2A).

In further experiments examining the invasiveness of tumor cells, we found that parental and control vector-transfected tumor cells efficiently penetrated the Matrigel-coated membrane, whereas the penetration rate of EBP1-transfected tumor cells was significantly reduced (Figure 2B).

To explore the mechanism of anti-invasiveness action of EBP1, we detected the effect of EBP1 on the expression of invasion-related factors. Extracellular matrix remodeling proteinases such as matrix metalloproteinases (MMPs) play a principal role in altering the local microenvironment during cancer invasion and distant spread [18]. MMPs expression in salivary gland cancer has thus been widely studied, with important findings that high MMP9 index in ACC was associated with poor survival [19-21]. Most recently a study suggested that epithelial-mesenchymal transition (EMT) led to loss of E-cadherin and gain of vimentin that induces tumor cell dissemination from the primary tumor site [22]. Therefore, we compared these molecules in vector or EBP1 stably-transfected ACC-M cell lines. Immunoblot analysis showed that stable transfection with EBP1 cDNA significantly reduced expression of MMP9 but boosted ICAM-1 and E-cadherin protein levels in ACC cells (Figure 2C and D).

### Correlations between EBP1 and MMP9, ICAM-1 and E-cadherin in ACC patients

Since we had shown that EBP1 regulates the protein levels of MMP9, E-cadherin and ICAM-1, we detected the immunoreactivity of MMP9, E-cadherin and ICAM-1 in the same 132 paraffin-embedded normal adjacent and carcinoma tissues as used for immunostaining of EBP1, then analyzed their correlations to better understand the pathophysiological and clinical context in which EBP1 might operate. As shown in Table 3, higher EBP1 expression was positively correlated with E-cadherin level *(P*<0.001) but negatively correlated with MMP9 expression (*P*=0.0002). EBP1 has been shown to constitutively activate ICAM-1 transcription [23], and our current results clearly demonstrated an extensive decrease of ICAM-1 expression in 50/66 ACC tissues. However, statistical analysis shows no negative correlation between EBP1 and ICAM-1 based on our current sample size.

**Table 3:** Correlation between Ebp1 and MMP9, ICAM-1 and E-cadherin immunostaining intensity in ACC tissues

**Variables**	**N (%)**	**Ebp1 expression**	**+**	**++**	**+++**	**p-value**
		**-**				
MMP9						
-	1	1	0	0	0	0.0002
+	1	0	0	1	0	
++	23	3	1	3	16	
+++	41	6	8	20	7	
ICAM-1						
-	50	8	8	16	18	0.6192
+	16	2	1	8	5	
++	0	0	0	0	0	
+++	0	0	0	0	0	
E-Cadherin						
-	1	1	0	0	0	<.0001
+	24	8	6	10	0	
++	18	1	3	13	1	
+++	22	0	0	1	21	

### Survival analysis

**Figure 3:**
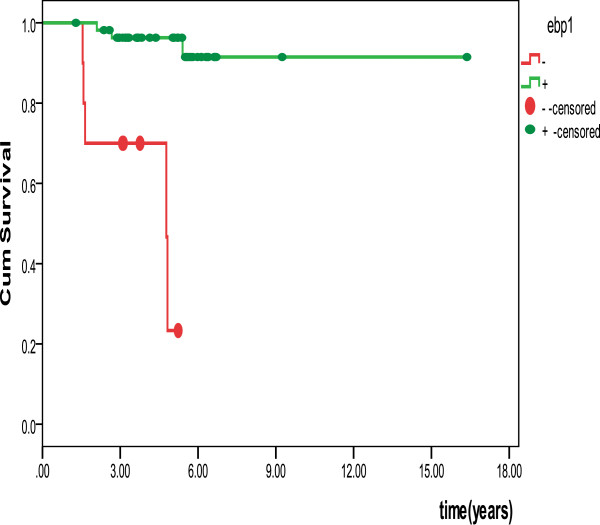
**Disease-free survival in cases with adenoid cystic carcinoma.** Disease-free survival of ACC patients was calculated by the Kaplan-Meier method. Patients with low or no EBP1 expression had significantly worse outcomes than patients showing higher EBP1 staining (*P*<0.0001, log rank test).

Kaplan-Meier survival curves are illustrated in Figure 3. Log rank tests indicated that a decrease in EBP1 expression was associated with a reduction in overall survival of patients with ACC (*P*<0.0001).

## Discussion

ACC is a relentless and unpredictable tumor with a tendency to invade perineural spaces, and is stubbornly recurrent. Eventually, 50% or more widely disseminate to distant sites such as bone and liver. Thus, although the 5-year survival rate is approximately 60%, it drops to 11% at 20 years [2,3], highlighting the importance of exploring the underlying molecules associated with recurrence and distant metastasis, and the significant obstacles for the cure of patients with ACC. For the first time, our current study profiled EBP1 expression in ACC patients and its clinicopathological relevance. Mainly, our results demonstrated that EBP1 expression was inversely correlated with the progression of ACC. This data is consistent with our previously published results showing that wild-type *EBP1* gene transfer into human salivary ACC cell line significantly inhibits cell proliferation in *in vitro* assays and reduces tumor metastatic potential in an animal model [11].

Histologically, ACC can be categorized into three types; tubular, solid and cribriform. The solid pattern is known to be much more aggressive than the other two types [24]. Our study showed that EBP1 expression is significantly higher in cases with tubular pattern than that with solid pattern. In line with the inhibitory property of the *EBP1* gene, as previously demonstrated in cancers of glandular epithelial origin such as prostate [8,12,25], breast [14] and salivary [11], our current findings may suggest, at least in part, that a decrease of EBP1 contributes to the more malignant behavior of solid type than tubular histotype. Interestingly, in ACC, EBP1 staining was localized predominantly to the cytoplasm of epithelial cells of glands, whereas in the adjacent non-cancerous tissues, abundant EBP1 immunoreactivity was observed in both cytoplasm and nuclear. Squatrito *et al.* found that both the N-terminal and the C-terminal regions of EBP1 are required for correct EBP1 localization, and that nucleolar localization is necessary for its growth suppression activity [26]. *EBP1* was reported to be mutated in 22% of patients with colorectal cancers [27]. We are thus poised to examine *EBP1* gene status to determine if EBP1 is a normal protein in ACC tissues that show strong positive EBP1 staining in the cytoplasm.

Insinuate perineural invasion and distant metastasis are characteristic of clinical features of ACC, and are the major challenge to very poor long-term outcome of patients with this disease [1,2,4]. Studies over more than 40 years revealed mounting evidence implicating matrix metalloproteinases (MMPs) to be the principal mediators in the initial proteolytic degradation of extracellular matrix (ECM) during cancer metastasis [18]. Elevated levels of MMPs have been associated with the invasive properties of various cancer types. In particular, high expression of MMP9 correlates with poor survival of ACC [19]. In this respect, MMPs might regulate cell-cell and cell-ECM interactions by processing E-cadherin and integrin, respectively, affecting both cell phenotype (EMT) and increasing cell migration [4,18]. Most recently, a report demonstrated that EMT with loss of E-cadherin and gain of vimentin induces ACC cells to break away from the primary tumor site, suggesting ACC uses unique mechanisms of invasion from those of other malignant tumors of the oral cavity [22]. Nevertheless, we found that EBP1 inhibits both motility and invasiveness of ACC cells, further supporting our previous findings [11]. Importantly, EBP1 downregulates MMP9 but enhances the protein levels of E-cadherin and another critical molecule, ICAM-1, which is involved in tumor immunity and metastasis. Since tumor attack by cytotoxic T lymphocytes and macrophages is mediated by the interaction of leukocyte function-associated antigen (LFA)-1 on lymphocytes with intercellular adhesion molecule (ICAM)-1 on the tumor surface [28,29], not surprisingly, reduced expression of ICAM-1 has been shown to promote immune evasion and metastasis, resulting in poor prognosis in patients with ACC [30].

Of note, reduced levels of EBP1 expression were significantly associated with perineural invasion and local lymphatic and distant lung metastasis of clinical adenoid cystic carcinoma. Wild-type EBP1 gene transfer into ACC-M cells led to reduced motility and invasiveness by suppressing MMP9 but enhancing protein levels of ICAM-1 and E-cadherin. Indeed, we found higher EBP1 expression was positively correlated with E-cadherin level but negatively correlated with MMP9 expression. Moreover, patients with lower EBP1 had a poorer long-term survival than those with positive EBP1 expression. Therefore, EBP1 might be a novel biomarker indicating local recurrence and distant metastasis, an unfavorable prognosis in ACC patients. It would be of great interest to further expand our studies by elucidating how EBP1 downregulates MMP9 but upregulates E-cadherin, given their therapeutic potential in ACC patients.

## Conclusion

EBP1 expression is reduced in ACC, indicating unfavorable prognosis of ACC patients. Assessment of EBP1 protein expression status in ACC patients by IHC will be useful in early detection and prognosis, and therefore in relevant clinical decision-making such as close monitoring as an alternative therapeutic modality against local invasion and recurrence.

## Abbreviations

EBP1: ERBB3 binding protein 1; ACC: Adenoid cystic carcinoma; IHC: Immunohistochemistry; LFA-1: Leukocyte function-associated antigen 1; ICAM-1: Intercellular adhesion molecule 1; EMT: Epithelial-mesenchymal transition; MMPs: Matrix metalloproteinases; ECM: Extracellular matrix.

## Competing interests

Authors declare that they have no competing interests.

## Authors’ contributions

YY: Study design, interpretation of the results and preparation of the manuscript for publication. JS, YL, ZT, LG, SX were responsible for performing immunohistochemistry analysis and other experiments, data collection and interpretation of the results. Pathologists JS, YL, ZT did histological examination, grading and evaluation of immunostaining. JS, LG, SX conducted statistical analysis. All authors read and approved the final manuscript.

## Pre-publication history

The pre-publication history for this paper can be accessed here:

http://www.biomedcentral.com/1471-2407/12/499/prepub
